# Cu_2_O-Based Electrochemical Biosensor for Non-Invasive and Portable Glucose Detection

**DOI:** 10.3390/bios12030174

**Published:** 2022-03-14

**Authors:** Fabiane Fantinelli Franco, Richard A. Hogg, Libu Manjakkal

**Affiliations:** 1 Water and Environment Group, Infrastructure and Environment Division, James Watt School of Engineering, University of Glasgow, Glasgow G12 8LT, UK; fabiane.fantinellifranco@glasgow.ac.uk; 2 Electronic and Nanoscale Engineering, James Watt School of Engineering, University of Glasgow, Glasgow G12 8LT, UK; richard.hogg@glasgow.ac.uk

**Keywords:** glucose sensor, Cu_2_O nanomaterial, electrochemical sensor, non-enzymatic sensor

## Abstract

Electrochemical voltammetric sensors are some of the most promising types of sensors for monitoring various physiological analytes due to their implementation as non-invasive and portable devices. Advantages in reduced analysis time, cost-effectiveness, selective sensing, and simple techniques with low-powered circuits distinguish voltammetric sensors from other methods. In this work, we developed a Cu_2_O-based non-enzymatic portable glucose sensor on a graphene paste printed on cellulose cloth. The electron transfer of Cu_2_O in a NaOH alkaline medium and sweat equivalent solution at very low potential (+0.35 V) enable its implementation as a low-powered portable glucose sensor. The redox mechanism of the electrodes with the analyte solution was confirmed through cyclic voltammetry, differential pulse voltammetry, and electrochemical impedance spectroscopy studies. The developed biocompatible, disposable, and reproducible sensors showed sensing performance in the range of 0.1 to 1 mM glucose, with a sensitivity of 1082.5 ± 4.7% µA mM^−1^ cm^−2^ on Cu_2_O coated glassy carbon electrode and 182.9 ± 8.83% µA mM^−1^ cm^−2^ on Cu_2_O coated graphene printed electrodes, making them a strong candidate for future portable, non-invasive glucose monitoring devices on biodegradable substrates. For portable applications we demonstrated the sensor on artificial sweat in 0.1 M NaOH solution, indicating the Cu_2_O nanocluster is selective to glucose from 0.0 to +0.6 V even in the presence of common interference such as urea and NaCl.

## 1. Introduction

Portable sensors are receiving significant and growing interest in healthcare management, especially for monitoring chronic diseases such as diabetes and chronic wounds. It has been noted that one of the major causes of mortality is diabetes related diseases, affecting 537 million people in 2021, a number that is expected to significantly grow in the coming years [1,2]. The increasing glucose levels in the world population denote the importance of developing new sensors which can monitor glucose levels in a cost-effective and simple manner. The present generation of glucose monitoring devices are minimally invasive and measure real-time interstitial fluid glucose levels; however, they still rely on skin piercing, with many patches lasting up to 10 days or less [3]. Other common methods include measurement of blood glucose by finger pricking or blood tests, which is inconvenient and painful [4]. Although these blood-based monitoring systems are well established and frequently used, not all diabetic patients comply with the protocol due to the pain and inconvenience associated with the invasive detection process [5]. As diabetes affects a large share of the population, new non-invasive methods, such as portable or wearable systems, are being extensively researched to increase the level of comfort in patients. As such, market growth for non-invasive glucose-monitoring devices is expected to reach USD 11.35 million between 2021 and 2025 [6]. However, most non-invasive devices incorporate complicated technologies that rely either on spectroscopy or optical techniques specifically tailored to a particular type of diabetes and/or a specific age group [6]. Therefore, cheap and reliable non-invasive techniques are still needed, especially to enhance the viability of routine glucose checkups and other applications in low-income and hard-to-reach areas. In this manner, electrochemical paper-based monitoring devices offer an opportunity for non-invasive detection by using biological fluids other than blood. They have the potential to generate robust, sensitive methods for the detection of metabolic changes in the medium and short term. However, the glucose pathway from blood to sweat has yet to be fully clarified [7]. There are also limitations in accuracy and sensitivity to environmental factors. As a result, the development of proper sampling techniques is urgently needed in order to estimate blood glucose via sweat. Recently, wearable electronics have begun to address these shortcomings with the development of integrated sensor arrays [8,9,10,11]. Electrochemical sensors offer the possibility of miniaturized, low-cost, portable sensors that require fewer reagents and no specialized personnel to operate them. Furthermore, the sensing range, cross-sensitivity, and stability can be improved by modifying the sensitive material.

In conventional electrochemical biosensors, the glucose oxidase enzyme is used to detect glucose in physiological pH conditions, since it provides good selectivity and sensitivity. However, enzymes are sensitive to changes in pH, temperature, and humidity, as well as interference from some electro-oxidizable reagents [5]. To overcome the issues due to the enzymatic process, metal and metal-oxide-based glucose sensors have been developed [12,13,14]. These are usually based on a composition of noble metals (e.g., Au and Pt) [15,16,17], transition metals (e.g., Cu, Ni, Zn, and Co) [18,19,20], metal-oxides (e.g., CoO, NiO, and CuO/Cu_2_O) [21,22], and their combination with carbon materials [14]. In particular, Cu-based glucose sensors have attracted attention as Cu is a low-cost material and has a wide crustal abundance. Moreover, Cu_2_O is a stable Cu oxide, and a p-type semiconducting material with a 2.17 eV bandgap, making it a versatile material for various applications, including solar cells, sensors, and batteries [23,24,25,26]. Its low net surface charge prevents the material from being affected by interference from other compounds that commonly affect noble metals [14]. The change of oxidation state from Cu(II) to Cu(III) mediates the electrocatalytic activity of copper nanocomposites, with the possibility of tailoring the synthesis to form nanostructures such as nanoflowers, nanowires, and nanocubes [27,28,29]. Therefore, Cu_2_O is a suitable non-enzymatic alternative for glucose sensing. For the fabrication of single-use biosensors, cellulose-based substrates are non-toxic and provide enhanced biocompatibility and biodegradability [30,31,32]. It is the most naturally abundant material in the form of wood and cotton, among others [33]. The highly porous structure and large surface area of the cellulose make it a sensible choice as a substrate for electrochemical biosensors.

**Figure 1:**
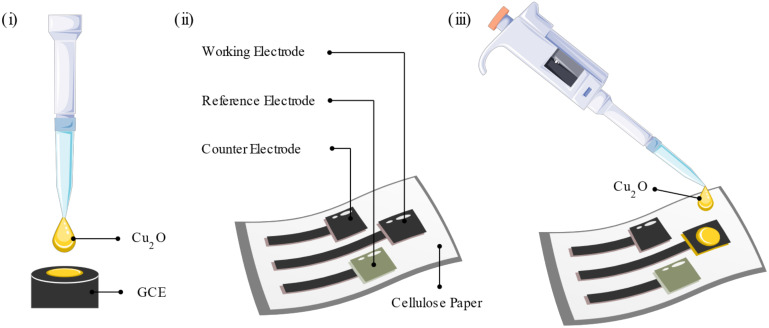
Schematic fabrication of the glucose sensor. (**i**) Cu_2_O nanoclusters drop casted on the GCE for material study. (**ii**) Graphene paste printed on cellulose cloth with a Ag/AgCl RE. (**iii**) Modified WE with Cu_2_O on the graphene printed cellulose substrate.

In this work, a non-invasive, portable sweat-based glucose sensor was fabricated by hand printing graphene paste electrodes on sustainable biodegradable and biocompatible cellulose substrates. Cu_2_O nanoclusters were employed as the sensitive material and drop casted on top of the working electrode (WE) to complete the affordable, voltammetric glucose sensors. To study the feasibility of the Cu_2_O nanoclusters for glucose sensing, cyclic voltammetry (CV), differential pulsed voltammetry (DPV), and electrochemical impedance spectroscopy (EIS) studies were conducted in 0.1 M sodium hydroxide (NaOH) and artificial sweat/NaOH solutions using Cu_2_O a coated glassy carbon electrode (GCE) or graphene paste printed electrodes (PEs). Commercial graphene paste was used for the printed electrodes, Ag/AgCl commercial paste was used for the pseudo-reference electrode (RE), and the WE was further modified with drop casted Cu_2_O nanoclusters. The schematic fabrication is shown in Figure 1. As a proof of concept to validate these portable glucose sensors, voltammetric sensors were tested in 0.1 M NaOH and artificial sweat/NaOH solutions with varying concentrations of glucose. X-ray diffraction (XRD) and scanning electron microscopy (SEM) were employed to study the morphology of the Cu_2_O nanoclusters and the interface of the sensitive material with the printing pastes and substrate. These biocompatible disposable and reproducible sensors showed good sensing performance in the range of 0.1 to 1 mM glucose, with a sensitivity of 1082.5 ± 4.7% µA mM^−1^ cm^−2^ on the GCE and 182.9 ± 8.83% µA mM^−1^ cm^−2^ on graphene PEs, making them a strong candidate for portable, non-invasive sweat glucose monitoring devices.

## 2. Materials and Methods

### 2.1. Cu_2_O Synthesis

The Cu_2_O nanocrystals were synthesized by modifying a previously published work [28]. Briefly, the synthesis consists of an ascorbic acid (C_6_H_8_O_6_) and NaOH reduction route at room temperature. The addition of C_6_H_8_O favors the formation of Cu_2_O, and the concentration of NaOH dictates the nanoparticle shape [34]. The reaction mechanism is as follows [34]:

2Cu(OH)_2_ + C_6_H_8_O_6_ → Cu_2_O + C_6_H_8_O_6_ + 3H_2_O (1)

Firstly, 0.1 mmol of CuCl_2_ and 0.1 g of polyvinylpyrrolidone were dissolved in 40 mL. After a dropwise addition of 2.5 mL of 0.2 M NaOH aqueous solution, the mixture was stirred for 5 min. Then, 2.5 mL of 0.1 M aqueous ascorbic acid was added dropwise, and the solution was stirred for further 5 min. The Cu_2_O crystals were recovered by centrifugation and washed two times with ethanol. The crystals were dried and suspended in deionized water (1 mg/mL) to be used for further experiments.

### 2.2. Sensor Fabrication

For the initial experiments, the sensitive electrode was fabricated on the top of the glassy-carbon electrode (GCE). For this, 5 µL of the Cu_2_O aqueous solution (1 mg/mL) was drop casted on the GCE and dried at 80 °C for 5 min. For the printed sensor fabrication, first a three-electrode layer was hand printed on top of a cellulose substrate using a graphene paste (JESC-7771G, JE Solutions Consultancy, UK). The RE and the CE were 3 mm × 1.5 cm lines, and the WE consisted of a 3 mm × 2 cm line and a 1 × 0.5 cm rectangle. Among these three electrodes, one was employed as a CE, and the surface of the second electrode was converted to a RE. For RE fabrication, a silver/silver chloride (Ag/AgCl) paste (JESC-7713AgCl, JE Solutions Consultancy, London, UK) was hand printed on the top of graphene electrode and heat treated at 80 °C for 1 h in the oven. Wires were attached to the contact pads using graphene paste and dried at 80 °C for 30 min. Before use, the WEs were further modified by drop casting 20 µL of the Cu_2_O aqueous solution and dried at 80 °C for a few minutes. Finally, the contact pads were covered with insulative tape, and only the active area of sensor was exposed to the solution.

### 2.3. Material Characterization

The structural characterization of the sensitive electrode was carried out with an X-Ray diffractometer (XRD, P’Analytical X‘Pert with Cu Kα (λ = 1.541 Å)). The morphological characterization and the atomic composition of the electrodes alongside the energy-dispersive X-ray spectroscopy (EDS, Oxford Instruments Energy 250) mapping were performed using a scanning electron microscope (SEM, Philips/FEI XL30 ESEM at 20 kV). The SEM images were analyzed using the ImageJ software.

### 2.4. Electrochemical Characterization

For electrochemical measurements all reagents were used as received and all solutions were prepared in deionized (DI) water unless otherwise mentioned. The CV, DPV, and EIS analyses of the sensors were carried out using a Gamry potentiostat (Interface 1010E). A three-electrode system, with a commercial glass Ag/AgCl RE, a platinum counter electrode (Pt CE), and a standard GCE, was employed in the initial material studies. A PE with drop casted Cu_2_O (as WE) with a commercial RE and CE was used for initial testing of the printed material, and the full printed sensor was employed for the final tests. The experiments were performed under normal ambient conditions. The CV and DPV electrochemical measurements were carried out in 0.1 M NaOH (pH 13) aqueous solution or artificial sweat alkaline solution (15 mM sodium chloride (NaCl), 3 mM potassium chloride, (KCl), and 22 mM urea in 0.1 M NaOH with a final pH of 13) with a glucose concentration varying from 100–1000 µM with a 100 or 300 µM glucose stepwise addition. The limit of detection (LOD) was calculated by using the standard deviation (SD) of the lowest calculation and the calibration slope (S). The equation used was LOD = (SD/S) ∗ 3. The EIS analysis was carried out with 0.1 mM glucose in 0.1 M NaOH from 100 kHz to 0.1 Hz.

## 3. Results and Discussion

### 3.1. Material Characterization

**Figure 2:**
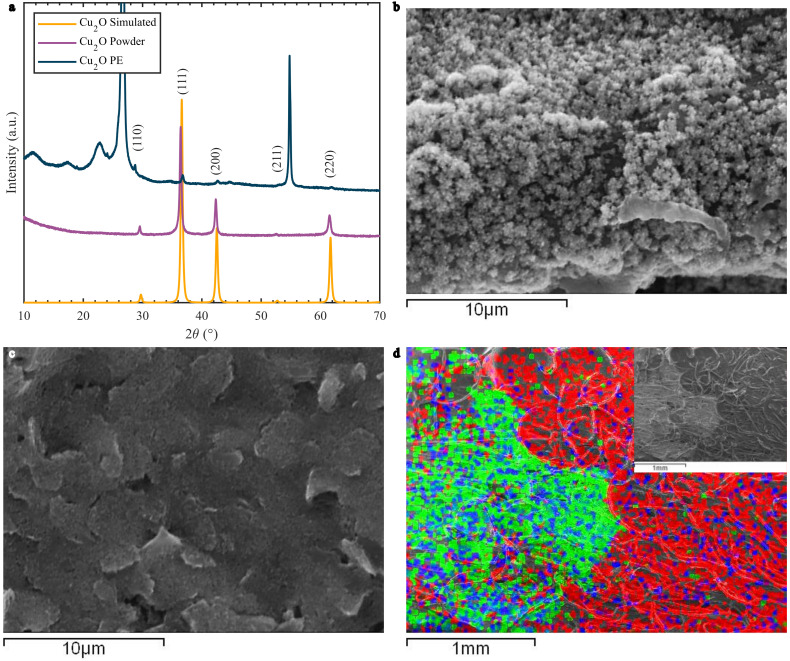
(**a**) XRD spectrum of simulated Cu_2_O, synthesized powder Cu_2_O, and WE drop casted with Cu_2_O nanoclusters (Cu_2_O PE). (**b**) SEM images of the Cu_2_O drop casted on the PEs: (**b**) Cu_2_O nanoclusters, (**c**) graphene paste and (**d**) EDS mapping of the interface between Cu_2_O and the graphene paste with an inset of the SEM image. Green corresponds to Cu, blue to O, and red to C.

The XRD spectra and the SEM images for the Cu_2_O materials and PEs are presented in Figure 2. The XRD spectra of the Cu_2_O synthesized powder and the Cu_2_O coated PE were compared to the simulated Cu_2_O spectrum (COD #96-100-0064), shown in Figure 2a. The powder and the Cu_2_O coated PE peaks matched the simulated cuprite data, indicating a successful synthesis. The Cu_2_O PE peaks were much less intense due to the low amount of drop casted Cu_2_O in relation to the surface of the WE. Some peaks were also slightly shifted to the right, indicating a change in microstructure parameters such as crystallite size and strain. This could be due to cuprite interaction with the graphene paste and agglomeration when drop casting. The Cu_2_O coated PE XRD spectrum also presented some new peaks from other crystalline materials present in the graphene paste and the substrate. Figure S1a displays the cellulose substrate and the PE with and without the drop casted Cu_2_O for comparison. The cellulose contribution is most prominent around 10 < 2θ < 28° due to the influence of its intra- and intermolecular bonding patterns on the cellulose polymer chain [30]. The other intense peaks correspond to the (002) and (004) peaks of graphite in the graphene paste [35].

The morphological structure of the Cu_2_O nanoclusters drop casted on the PEs can be seen in Figure 2b–d. The Cu_2_O nanomaterial appears to form clusters on top of the graphene-paste-based electrode. These clusters are a few micrometers in size and closely dispersed (Figure S2a). The graphene paste adhered well on the cellulose substrate, forming tightly packed layers. This is due to the fibrous, porous structure of cellulose, shown in Figure S2b, which facilitates strong adhesion of materials to its surface. From the EDS mapping (Figure 2d), a clear interface from the drop casted Cu_2_O crystals and the graphene paste can be observed, indicating where the cupric material dried and clustered. The EDS spectrum (Figure S2c) showed the presence of Cl, C, O, and Cu on the drop casted site. This could indicate that not all CuCl_2_ was washed and remained in the final suspension. The graphene paste has odd spots of Cu that could have appeared due to splatters or material run off. On the Cu_2_O side, both Cu and O are present, with oxygen sites exposed to the surface. This facilitates the formation of hydroxyl groups and the CuOOH configuration, responsible for the glucose oxidation process [29].

### 3.2. Cu_2_O Nanocluster Study on GCE

**Figure 3:**
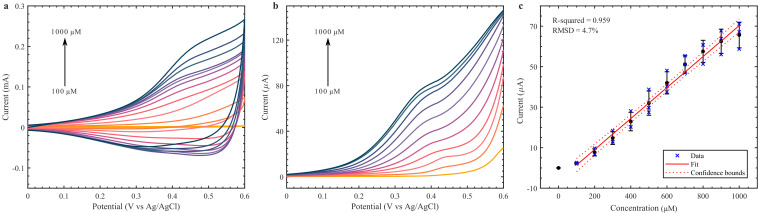
CV (**a**) and DPV (**b**) of Cu_2_O nanoclusters on GCE with varying glucose concentrations (100–1000 µM) in 0.1 M NaOH. (**c**) Calibration curve at 0.35 V from DPV analysis.

Firstly, the Cu_2_O nanoclusters were studied with a standard three-electrode configuration by drop casting the material on a commercial GCE. The material was utilized as an enzyme-free glucose detection method, and its electrochemical activity was characterized by CV and DPV on 0.1 M NaOH from 0.0 to +0.6 V, shown in Figure 3. This potential range is commonly employed in the detection of glucose by Cu-based materials [21,29]. The Cu material showed a clear redox response with the addition of glucose, from 100 to 1000 µM. Glucose concentration in sweat is significantly lower than blood glucose, ranging from 10 to 1100 µM [8]. Therefore, the Cu_2_O nanoclusters seem suitable for sweat applications. From the DPV (Figure 3b), an increase in peak current can be observed with every 100 µM addition of glucose. By setting the peak current at +0.35 V over three sets of drop casted Cu_2_O GCEs, a calibration curve was acquired (Figure 3c). A sensitivity of 1082.5 µA mM^−1^ cm^−2^ with a root mean square deviation (RMSD) of ±4.7% and R^2^ = 0.959. The upper limit of detection seems to be 1000 µM, where the linear curve starts to stabilize. The calculated LOD was 12 µM.

We studied the electron transfer process of the Cu_2_O nanoclusters with 1 mM of glucose at 0.1 M NaOH by changing the CV scanning rate from 50 to 300 mV/s (Figure S3). The oxidation peak current of glucose increases with faster scanning rates and shifts to slightly more positive potentials. The positive potential shift indicates a slow electron transfer process, while the linear fit of the anodic and cathodic peak currents implies a surface diffusion-limited process [36] due to porous electrode surface as confirmed in SEM image in Figure 2b. This is in line with other literature reports for Cu_2_O/CuO-based glucose electrocatalysis in alkaline media [37,38]. The mechanism of glucose oxidation on cuprite is not fully understood, but it is assumed that the CuOOH oxidant reagent is responsible for the glucose oxidation into gluconolactone [29,39]. The reaction can be expressed as follows:

Cu_2_O + 2OH^−^ + H_2_O → 2Cu(OH)_2_ + 2e^−^ (2)

Cu(OH)_2_ → CuO + H_2_O (3)

CuO + OH^−^ → CuOOH + e^−^ (4)

CuOOH + e^−^ + glucose → CuO + OH^−^ + gluconolactone (5)

2CuO + H_2_O + 2e^−^ → Cu_2_O + 2OH^−^ (6)

Equations (2)–(6) show the importance of an alkaline medium in providing the necessary OH^−^ group for the oxidation process. The cuprite material was also validated in an artificial sweat solution in 0.1 M NaOH (Figure S5). The material presented a similar performance to that of 0.1 M NaOH, indicating that common substances found in sweat (urea, NaCl, KCl) did not affect the oxidation process.

### 3.3. Printed Glucose Sensor Characterization

**Figure 4:**
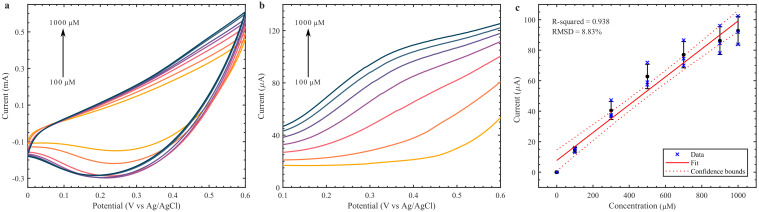
CV (**a**) and DPV (**b**) of Cu_2_O nanoclusters on printed WE with varying glucose concentrations (100–1000 µM) in 0.1 M NaOH. (**c**) Calibration curve at 0.35 V from DPV analysis.

The performance of the printed glucose sensors was initially validated using the Cu_2_O coated PEs with commercial RE and CE. The measured CV and DPV for various concentrations of glucose in 0.1 M NaOH solution are given in Figure 4a,b respectively. We noted that the peaks in CV and DPV were not as clear in the Cu_2_O coated on PE as compared to the Cu_2_O coated on top of GCE. Although the peaks were not as clear, the Cu_2_O PE could still be calibrated at +0.35 V. The calibration curve for three Cu_2_O PEs is seen in Figure 4c, with a sensitivity of 182.9 ± 8.83% µA mM^−1^ cm^−2^ and R^2^ = 0.938 and a calculated LOD of 52.7 µM. As the material was drop casted, it is difficult to calculate the effective area of the electrode, so the total graphene WE area was used in the calculation even though the Cu_2_O did not cover the whole area. This could underestimate the area sensitivity of the printed electrode, explaining the lower value. The calibration curve was obtained by subtracting the base current without the addition of glucose to each concentration point. Interestingly, this improved the calibration performance of the Cu_2_O PE but only marginally improved it for the GCE (Figure S4). While the R^2^ went from 0.843 to 0.938, and the RMSD from 14.9% to 8.83% for the Cu_2_O PEs, the GCE only saw a change of 0.01 for the R^2^ and 0.09% for the RMSD. This indicates that the baseline current is more significant for the PEs than for the GCE, and a correction is necessary to lower the error between different electrodes.

**Figure 5:**
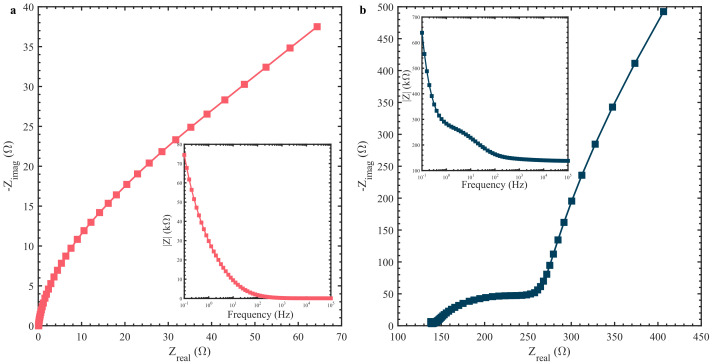
Nyquist plot of 0.1 M NaOH with 100 µM glucose solution of (**a**) Cu_2_O coated GCE with magnitude of impedance inset and (**b**) Cu_2_O coated on PE with magnitude of impedance inset.

The measurement shows that both the CV and DPV peaks were not as defined as on the GCE. This variation in performance could be due to the influence of the graphene printed electrode with the solution and less surface area available due to the agglomeration of the Cu_2_O on the surface of the PE as observed in Figure 2b. Further investigation of the influence of electrodes on the electrochemical Cu_2_O response was carried out using EIS analysis. Figure 5a shows the complex impedance data of Cu_2_O coated GCE through the Nyquist and Bode impedance magnitude plot (inset of the Figure 5a) for 0.1 M NaOH with a 100 µM concentration of glucose. For a similar solution, the impedance data plot for Cu_2_O coated on PE is shown in Figure 5b. The impedance data on the Nyquist plot shows that both GCE- and PE-based electrodes have an almost straight line in low frequency range. This could be due to the diffusion controlled reaction given in Figure S3b. However, we noted that the Cu_2_O coated GCE has a very high impedance value when compared to the low impedance presented by the PE. It reveals that the electrolyte distribution on the surface of the PE electrode is much better than on the GCE, leading to a lower resistance of the solution reaction with the electrode. Moreover, both the pseudo-capacitance of Cu_2_O and the electrochemical double layer capacitance from the printed graphene electrode contribute to the electrochemical properties of electrodes. The diffusion of ions in the bulk of the printed electrode can be confirmed from the small semicircle arc in the high frequency range in Figure 5b. In the GCE-based electrode the redox reaction due to the Cu_2_O material is more prominent, which can be observed by the distinguishable redox reaction in Figure 3a as opposed to the quasi-rectangle CV curve on the PE in Figure 4a. This difference in electrode–electrolyte reaction while coating on GCE or PE causes the variation in sensitivity of both sensors.

**Figure 6:**
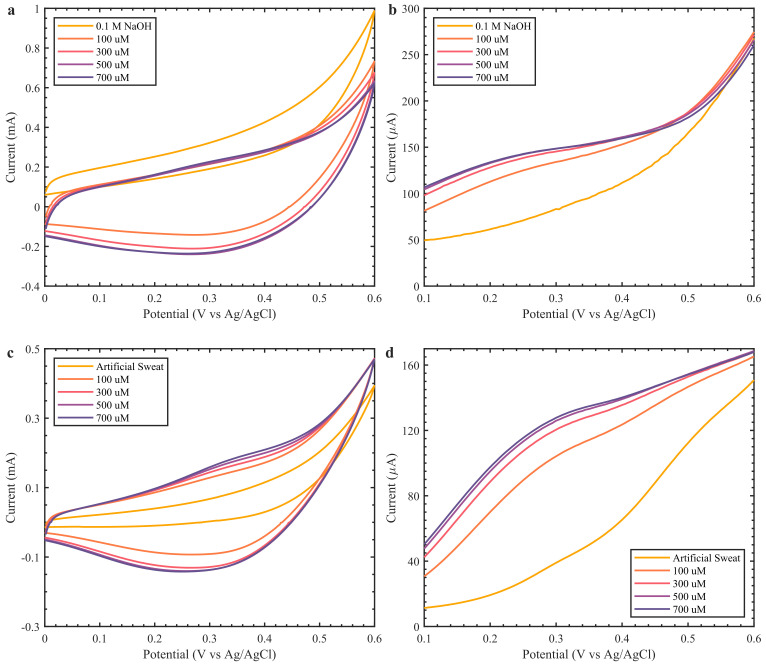
Electrochemical analysis of fully printed Cu_2_O-based glucose sensors over a glucose concentration range of 100–700 µM. (**a**) CV and (**b**) DPV in 0.1 M NaOH. (**c**) CV and (**d**) DPV artificial sweat in 0.1 M NaOH.

Finally, for a portable application we tested the performance of fully printed sensors (Figure 1(iii)) in 0.1 M NaOH and artificial alkaline sweat (Figure 6). The CV and DPV for fully printed sensors in 0.1 M NaOH solution with various glucose concentrations are given in Figure 6a,b. The CV and DPV performance of the sensor in artificial sweat with 0.1 M NaOH is given in Figure 6c,d. The preliminary investigation showed an increase in current up to 500 µM in both media. However, the artificial sweat in 0.1 M NaOH presented more defined peaks and stable oxidation peaks, around the same potential as that observed in previous results, while in 0.1 M NaOH solution the peaks shifted to the left. This could be the influence of the pseudo-Ag/AgCl RE, as the artificial sweat solution provides Cl^−^ ions to replenish the salt layer, while the NaOH does not provide the same favorable conditions. Although the preliminary results are promising, further investigation is needed to fabricate reliable fully printed glucose sensors that can support a wider range of glucose concentrations. The printed sensor performance was compared to other printed CuO/Cu_2_O-based glucose sensors (Table 1). It can be observed that the fabricated PE-based sensor is comparable to other reported works. Moreover, the low cost of fabrication, biocompatible substrate and electrodes, and biodegradable materials are the major advantages. The sensor size and biodegradable and sustainable textile-based substrate together with its facile fabrication steps facilitate its implementation as a portable glucose sensor.

## 4. Conclusions

Non-invasive, portable glucose sensors are necessary for increasing the wellbeing of patients living with diabetes. In this paper, we introduce a disposable non-enzymatic Cu_2_O-based sensor for portable glucose detection. We synthesized Cu_2_O crystals using a simple ascorbic acid reduction route and studied the material performance for glucose detection. The Cu_2_O formed nanoclusters and stayed on the surface of the graphene paste, with oxygen exposed to the surface. The Cu_2_O nanomaterial showed good performance towards glucose detection in basic medium, with a sensitivity of 1082.5 µA mM^−1^ cm^−2^ on GCE and 182.9 ± 8.83% µA mM^−1^ cm^−2^ at +0.35 V on graphene PEs. The RMSD was comparably low even on the printed sensors, indicating suitability for disposable sensors. Both the Cu_2_O on GCE and Cu_2_O PEs demonstrated a similar performance in the artificial sweat in 0.1 M NaOH solution, indicating that the Cu_2_O nanocluster is selective to glucose from 0.0 to +0.6 V even in the presence of common interference such as urea and NaCl. To assess their suitability for portable glucose detection, fully printed sensors were tested in 0.1 M NaOH and artificial sweat. The sensors showed a similar performance to the Cu_2_O PEs, supporting that these cheap, biodegradable sensors can be employed as portable disposable glucose sensors. However, drop casting the Cu2O material on the printed sensitive electrodes caused a decrease in sensitivity due to poor material dispersion and adhesion. By further improving the Cu_2_O nanomaterial and testing new techniques for better adhesion of the sensitive material on the carbon electrode, e.g., screen-printing and inkjet printing, these sensors could be employed in portable glucose sensing and hard-to-reach zones to simplify glucose monitoring.

## Figures and Tables

**Table 1:** Comparison of CuO/Cu_2_O-based non-enzymatic electrochemical glucose sensors.

Electrode Material	Substrate	Sensitivity (µA mM^−1^ cm^−2^)	Linear Range (mM)	Applied Potential (V)	LOD (µM)	Reference
CuO nanofibers	GCE	431.3	0.006–2.5	0.4	0.8	[39]
Cu_2_O nanocubes	SPCE	1040	0.007–4.5	0.7	31	[23]
Cu_2_O nanocubes/nafion	GCE	2864	0.05–5.65	0.7	1.7	[40]
Cu_2_O NPs/nafion	GCE	190	0.05–1.1	0.5	47.2	[41]
Cu_2_O nanowires	Cu foil	4060	0.001–2.0	0.55	0.58	[25]
Cu_2_O nanowires	Cu foam	6680.7	0.001–1.8	0.5	0.67	[42]
CuO/Cu_2_O nanosheets	Cu foil	1541	0.001–4	0.6	0.57	[22]
Cu_2_O nanoclusters	GCE	1082.5	0.1–1	0.35 (DPV)	12	This work
Cu_2_O nanoclusters	Cellulose PE	182.9	0.1–1	0.35 (DPV)	52.7	This work

SPCE = screen printed carbon electrode; NPs = nanoparticles.

## Supplementary Materials

The following are available online at https://www.mdpi.com/article/10.3390/bios12030174/s1, Figure S1: XRD spectrum of cellulose substrate, graphene paste printed on the cellulose substrate (C-PE), and Cu_2_O PE; Figure S2: SEM images of the (a) Cu_2_O drop casted on the PEs and (b) cellulose substrate. (c) EDS spectrum of the Cu_2_O drop casted on the PE with the SEM image inset; Figure S3: (a) CV curves with changing scan rates (50 to 300 mV/s) of Cu_2_O GCE with 1 mM glucose in 0.1 M NaOH. (b) Relationship between peak current and square root of scan rate. Figure S4: Unmodified calibration curves (without subtracting the baseline) of (a) Cu_2_O GCEs and (b) Cu_2_O PEs; Figure S5: Unmodified calibration curves (without subtracting the baseline) of (a) Cu_2_O GCEs and (b) Cu_2_O PEs.
