# Supplementary material for: Cu2O-Based Electrochemical Biosensor for Non-Invasive and Portable Glucose Detection
Source: Biosensors (Basel). 2022 Mar 14;12(3):174. doi: 10.3390/bios12030174 (PMC8946795; doi:10.3390/bios12030174)
Supplement: Supplementary file 1 [file biosensors-12-00174-s001.zip › biosensors-1621239-supplementary.pdf]

Non-enzymatic Cu_2O-based Electrochemical Sensor for Non-Invasive Portable Glucose Detection

Fabiane Fantinelli Franco [1], Richard A. Hogg[2], Libu Manjakkal [2,*]

1 Water and Environment Group, Infrastructure and Environment Division, James Watt School of Engineering, University of Glasgow, Glasgow, G12 8LT, UK.; Fabiane.FantinelliFranco@glasgow.ac.uk

2 Electronic and Nanoscale Engineering, James Watt School of Engineering, University of Glasgow, Glasgow, G12 8LT, UK.; Richard.Hogg@glasgow.ac.uk; Libu.Manjakkal@glasgow.ac.uk;

* Correspondence: Libu.Manjakkal@glasgow.ac.uk;

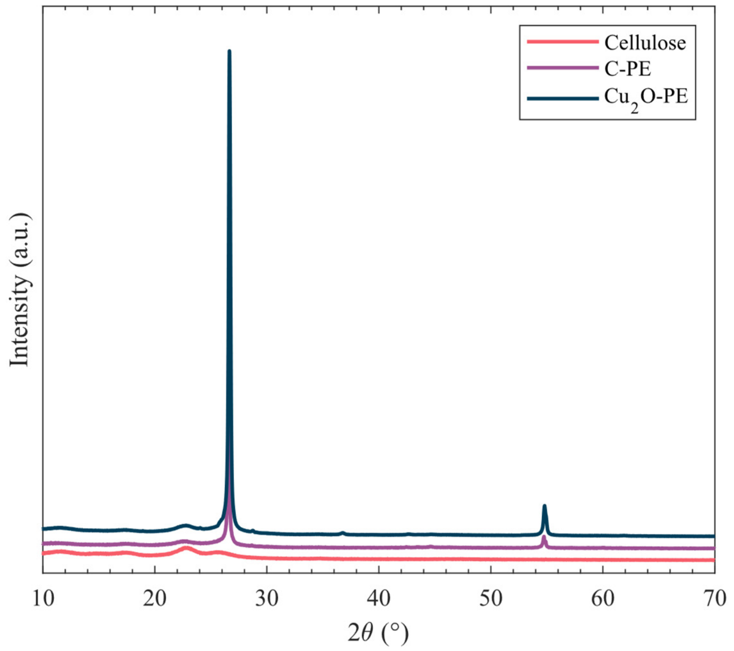

Figure S1. XRD spectrum of cellulose substrate, graphene paste printed on the cellulose substrate (C-PE), and Cu_2O PE.

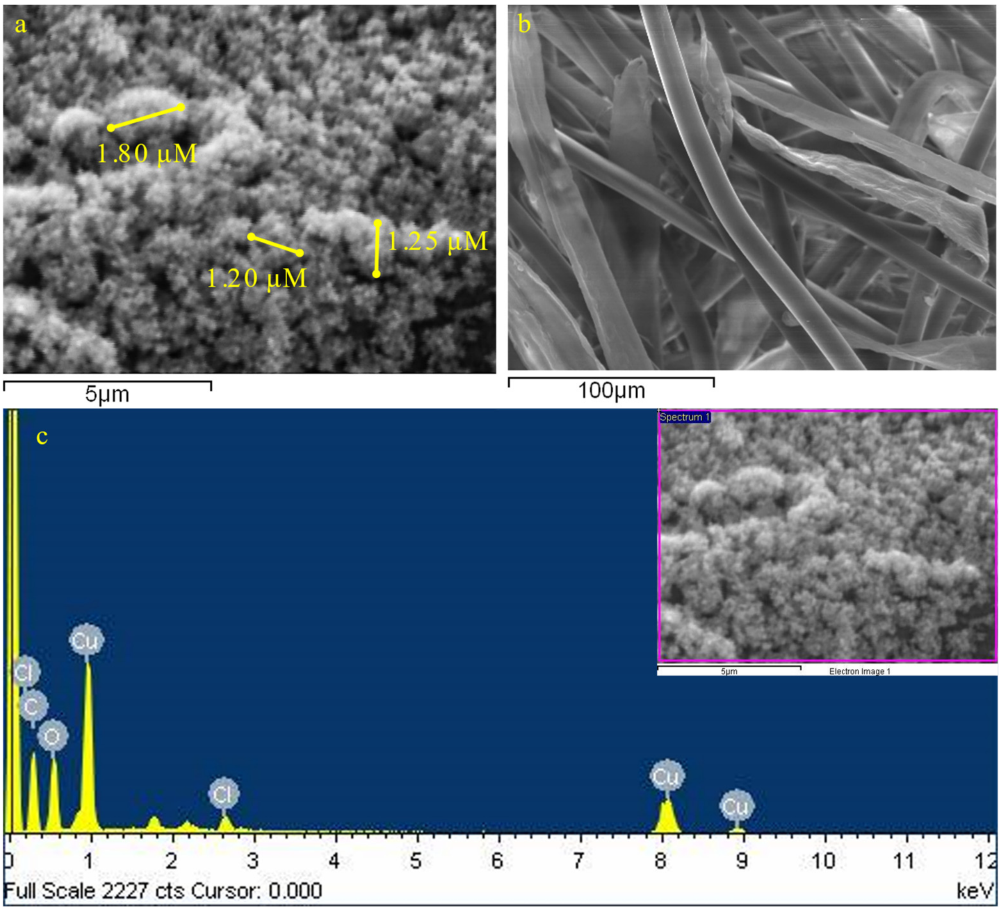

Figure S2. SEM images of the a) Cu_2O drop casted on the PEs and b) cellulose substrate. c) EDS spectrum of the Cu_2O drop casted on the PE with the SEM image inset.

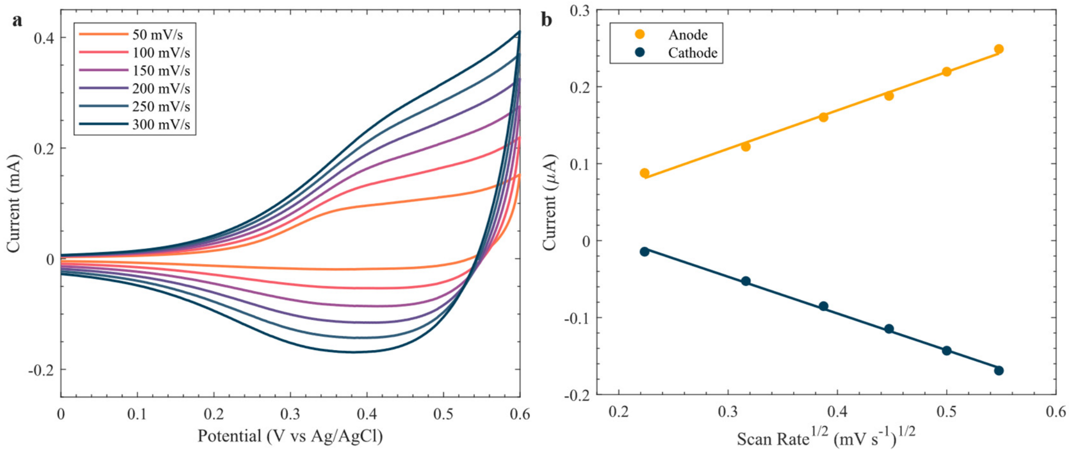

Figure S3. a) CV curves with changing scan rates (50 mV/s to 300 mV/s) of Cu_2O GCE with 1 mM glucose in 0.1 M NaOH. b) Relationship between peak current and square root of scan rate.

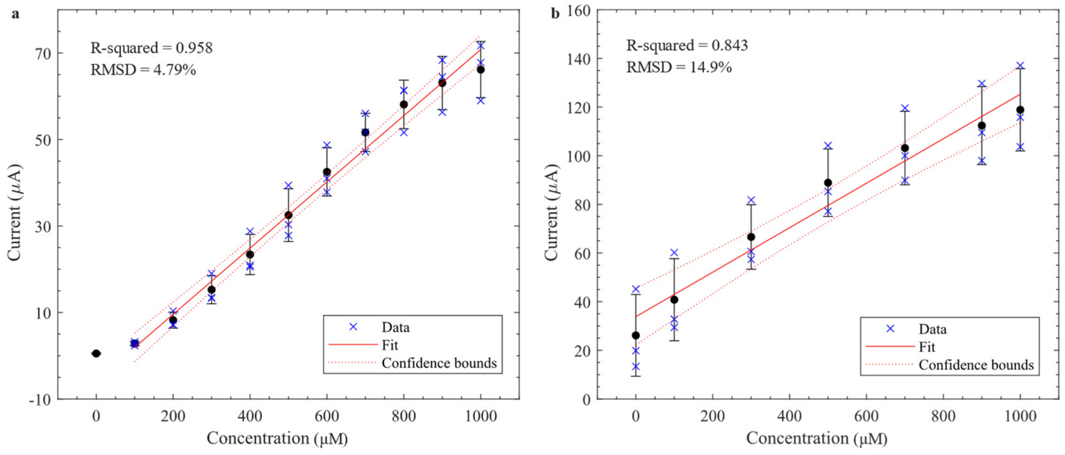

Figure S4. Unmodified calibration curves (without subtracting the baseline) of a) Cu_2O GCEs and b) Cu_2O PEs.

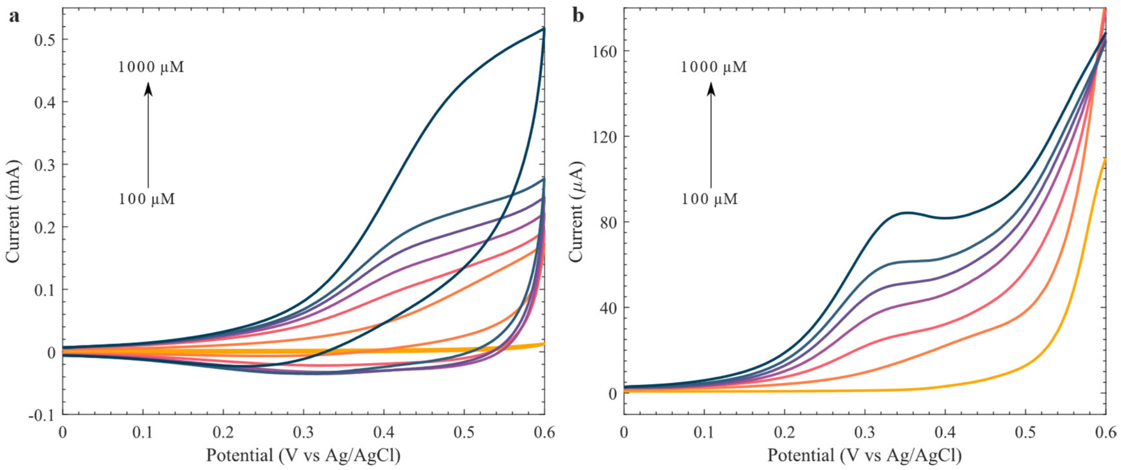

Figure S5. Unmodified calibration curves (without subtracting the baseline) of a) Cu_2O GCEs and b) Cu_2O PEs.
